# Replication and Oncolytic Activity of an Avian Orthoreovirus in Human Hepatocellular Carcinoma Cells

**DOI:** 10.3390/v9040090

**Published:** 2017-04-24

**Authors:** Robert A. Kozak, Larissa Hattin, Mia J. Biondi, Juan C. Corredor, Scott Walsh, Max Xue-Zhong, Justin Manuel, Ian D. McGilvray, Jason Morgenstern, Evan Lusty, Vera Cherepanov, Betty-Anne McBey, David Leishman, Jordan J. Feld, Byram Bridle, Éva Nagy

**Affiliations:** 1 Department of Pathobiology, Ontario Veterinary College, University of Guelph, Guelph, ON N1G 2W1, Canada; rob.kozak@gmail.com (R.A.K.); larissa.hattin@medportal.ca (L.H.); corredor@uoguelph.ca (J.C.C.); scott.walsh22@gmail.com (S.W.); jason.d.morgenstern@gmail.com (J.M.); evanlusty@gmail.com (E.L.); bmcbey@uoguelph.ca (B.-A.M.); dleishma@uoguelph.ca (D.L.); bbridle@uoguelph.ca (B.B.); 2 Sandra Rotman Centre for Global Health, University of Toronto, Toronto, ON M5G 1L7, Canada; mia.biondi@mail.mcgill.ca (M.J.B.); vera.cherepanov@uhn.ca (V.C.); Jordan.feld@uhn.ca (J.J.F.); 3 Multi-Organ Transplant Program, Department of Surgery, University of Toronto, Toronto General Hospital, Toronto, ON M5G 2C4, Canada; maxuezhong@hotmail.com (M.X.-Z.); jmanuel@research.ca (J.M.); Ian.McGilvray@uhn.on.ca (I.D.M.)

**Keywords:** avian orthoreovirus, hepatocellular carcinoma, hepatitis C virus, oncolytic virus, syncytia

## Abstract

Oncolytic viruses are cancer therapeutics with promising outcomes in pre-clinical and clinical settings. Animal viruses have the possibility to avoid pre-existing immunity in humans, while being safe and immunostimulatory. We isolated an avian orthoreovirus (ARV-PB1), and tested it against a panel of hepatocellular carcinoma cells. We found that ARV-PB1 replicated well and induced strong cytopathic effects. It was determined that one mechanism of cell death was through syncytia formation, resulting in apoptosis and induction of interferon stimulated genes (ISGs). As hepatitis C virus (HCV) is a major cause of hepatocellular carcinoma worldwide, we investigated the effect of ARV-PB1 against cells already infected with this virus. Both HCV replicon-containing and infected cells supported ARV-PB1 replication and underwent cytolysis. Finally, we generated in silico models to compare the structures of human reovirus- and ARV-PB1-derived S1 proteins, which are the primary targets of neutralizing antibodies. Tertiary alignments confirmed that ARV-PB1 differs from its human homolog, suggesting that immunity to human reoviruses would not be a barrier to its use. Therefore, ARV-PB1 can potentially expand the repertoire of oncolytic viruses for treatment of human hepatocellular carcinoma and other malignancies.

## 1. Introduction

Liver cancer is the third leading cause of cancer worldwide, and hepatocellular carcinoma (HCC) represents approximately 70%–80% of all cases [1]. The incidence of HCC is expected to increase markedly in North America over the next decades due to the high prevalence of hepatitis C virus (HCV) infection [2]. Additionally, as the prevalence of obesity and type 2 diabetes rises, metabolic diseases related to HCC will also continue to contribute to this burden [1]. Despite studies into the pathogenesis of HCC, and attempts to enhance therapy, improvement in patient outcomes has been marginal. The use of oncolytic viruses (OVs) for therapy offers promise for cancers where survival outcomes are poor and treatment options are limited. The use of these viruses offers numerous advantages over conventional cancer therapies including reduced toxicity, treatments of relatively short duration, and the possibility of targeting micrometastases [3]. The list of candidates includes numerous DNA and RNA viruses [4,5,6,7] from a variety of viral families (extensively reviewed in [8,9]). Most recently, the oncolytic human herpes simplex virus 1 (HSV-1) carrying the granulocyte macrophage colony-stimulating factor (GM-CSF), known as T-VEC, was approved for melanoma treatment [10].

An ideal OV should eliminate cancer cells through a combination of three mechanisms: direct oncolysis, antiangiogenic or antivasculature effects, and activation of innate- and tumor-specific immune responses [8]. Currently, a number of human viruses are undergoing pre-clinical and clinical trials as OVs [11,12,13,14]. However, the presence of pre-existing neutralizing antibodies (such as those generated against human adenovirus serotype 5) reduces the potency of OVs. Even if a patient does not initially have pre-existing antibodies, they will be induced after treatment with an OV, thereby limiting the number of doses that can be administered [15]. Furthermore, while administration by alternative routes (e.g., intravenous) can be a potential means to circumvent this, it is highly likely that effective OV therapy will require multiple viruses, potentially in combination with chemotherapy or radiation. Animal-derived viruses that do not circulate extensively in the human population represent a potential source of OVs that can circumvent pre-existing immunity. Several animal viruses with demonstrated oncolytic properties, such as Newcastle disease virus, replicate and induce strong cytopathic effects (CPE) in human cancer cell lines [16].

Oncolytic animal viruses are often potent activators of the immune system, as their evolution in a non-human host has limited their ability to evade the immune response in humans [17,18]. Therefore, these viruses can potentiate immunocentric oncolytic virotherapy, where emphasis is placed on induction of immunological mechanisms that can target cancer cells [19]. Avian reoviruses (ARVs) are non-enveloped and some of them cause diseases in poultry. These viruses are part of the *Orthoreovirus* genus, and, although they share similarities with the mammalian reoviruses, they form a separate species, *Avian orthoreovirus*. In contrast to mammalian reoviruses, the receptor(s) for avian reoviruses has yet to be identified, although these viruses have a wide-range in terms of cellular tropism, as infections in poultry involve multiple systems and organs (including the liver), the most important disease in chickens is viral arthritis [20,21].

In general, reoviruses replicate in the cytoplasm of host cells, and their double-stranded (ds)RNA genome is composed of 10 segments [22]. Mammalian reoviruses are currently being investigated in the clinic and have demonstrated efficacy in conjunction with chemotherapy against head and neck cancer [23]. We postulate that ARVs offer several unique characteristics, and may provide a complementary platform for oncolytic virotherapy. First, ARVs are not known to be associated with human disease and pre-existing immunity would not hamper their clinical application. Second, unlike mammalian reoviruses, ARVs can induce syncytia through the fusion-associated transmembrane (FAST) protein [24,25,26] and thus it may facilitate virus spread and distribution within a tumor. Additionally, the ARV p17 protein induces autophagy through multiple pathways and activates protein kinase RNA-activated (PKR) signaling [27]. Both processes activate the innate immune system, which can induce immune responses against tumors.

In this work, we found that ARV-PB1 replicated and induced cytopathic effects in HCC cells. Mechanisms of cell death involved syncytia formation and apoptosis. Furthermore, ARV-PB1 induced the expression of interferon-stimulated genes (ISGs), which may be important in the induction of anti-tumoral immune responses.

## 2. Materials and Methods

### 2.1. Viruses

The original sample was obtained from the Animal Health Laboratory (AHL, University of Guelph, Guelph, ON, Canada) as a non-pathogenic serotype 11 fowl adenovirus (FAdV-11). Primary virus isolation at AHL was done from a chicken of a broiler flock presented with poorer performance and potential airsacculitis. Initially, the case was considered as FAdV associated inclusion body hepatitis (IBH), however the final diagnosis was non-IBH associated FAdV involvement. The presence and serotype of the isolated FAdV was demonstrated by polymerase chain reaction (PCR) and sequence analysis of the product, performed in the diagnostic lab. Isolation of more than one virus from chickens with performance issues is not uncommon. In this case, it is more the reovirus load in the tested tissues was very low and the subsequent passages in cell culture amplified the reovirus (ARV-PB1). The orthoreovirus ARV-PB1 (avian reovirus pathobiology 1) was initially detected in a field sample, and was subsequently isolated by multiple rounds of plaque purification in an avian hepatoma cell line (CH-SAH). Stocks of virus were generated in CH-SAH cells by successive freeze-thaw cycles and centrifugation. Virus propagation, titration and one-step growth curves were carried out in chicken hepatoma cells (CH-SAH cell line), as described [28]. To investigate the pre-existing immunity to ARV-PB1, plaque reduction assays were carried out using sera from HCC patients as described [29]. To assess the relative efficacy of ARV-PB1, it was compared to Reolysin^TM^ (Oncolytics Biotech Inc., Calgary, AB, Canada), a mammalian type 3 (Dearing strain) of reovirus, as a gold standard control, since it is undergoing extensive testing in human clinical trials (kindly provided by Dr. Matt Coffey, Oncolytics Biotech Inc.).

### 2.2. Cell Lines

Human and murine tumor cell lines were maintained in media supplemented with 10% fetal bovine serum (FBS), 2 mM l-glutamine, and penicillin (100 IU/mL)/streptomycin (100 μg/mL) (Sigma, Oakville, ON, Canada). The following cell lines were used: HepG2, Huh-7, Huh-7.5, Huh-7.5.1, BB7 as well as HeLa, L1210, PC-3, 22RV1, DU145, HCT 116, SK0V-3—obtained from the American Type Culture Collection (ATCC; Manassas, VA, USA)—and ID8 (kindly provided by Dr. James Petrik, University of Guelph, Guelph, ON, Canada). The CH-SAH cell line was maintained in DMEM/F12 (Dulbecco's Modified Eagle Medium: Nutrient Mixture F-12, Sigma) supplemented with 10% FBS, 2 mM l-glutamine, and penicillin (100IU/mL)/streptomycin (100 ug/mL).

### 2.3. Infection of Cells with Hepatitis C Virus (JFH-1 Strain)

Cells were seeded in 24-well plates and some of them were infected with JFH-1 virus (gift from Dr. Jake Liang, the National Institutes of Health (NIH), Liver Diseases Branch, Bethesda, MD, USA) at a multiplicity of infection (MOI) of 0.05 for 24 h. The cells were treated with ARV-PB1 at various MOIs, and after 48 h the cells were formalin-fixed and stained with crystal violet solution.

### 2.4. Preparation of Primary Hepatocytes

Human liver tissue was obtained from livers procured from multi-organ donors. The donor livers were perfused in situ with cold histidine-tryptophan-ketoglutarate (HTK) solution. The caudate lobe was resected and stored in HTK solution on ice before cell isolation. Briefly, the perfusion system consisted of a circulation pump which warmed the perfusion reagents to 41°C within a biological safety cabinet. Gas containing 95% O_2_:5% CO_2_ was used to oxygenate all reagents before perfuse into liver tissue. The average weight of the liver tissue was 10 to 20 g. Two or three irrigation cannulas with olive tips were inserted into the cut surface of the liver. Hank’s balanced salt solution (HBSS) was used to flush out remaining blood followed by HBSS containing 0.1 mM EGTA (ethylene glycol-bis(β-aminoethyl ether)-N,N,N',N'-tetraacetic acid) for 10 min. Subsequently, 250 mL of HBSS containing 0.5 µM calcium chloride was added followed by fluid with collagenase containing neutral protease (VitaCyte, Indianapolis, IN, USA) according to the manufacturer’s instructions. Perfusion was performed with recirculation of fluid for 15 to 20 min or until the liver appeared to break apart slightly under the Glisson’s capsule. The digested liver was then placed in a crystallizing dish containing 100–200 mL of HBSS with 0.1% human albumin. The tissue was then cut to release cells contained. Cells were filtered through a 210 μm nylon mesh followed by a 70 μm nylon mesh. Filtered cells were centrifuged at 72× *g* for 5 min at 4 °C. The hepatocyte cell pellet was washed twice as above and HBSS with 0.1% human albumin was added to re-suspend cells. Approximately 8–12 million viable cells per gram of tissue were isolated as determined by Beckman ViCell trypan blue system.

Primary hepatocytes thawed and transferred into Williams E Medium supplemented (Life Technologies, Burlington, ON, Canada) with 5% FBS, 1 µM DMSO (dimethyl sulfoxide) and thawing plating cocktail A (Life Technologies) according to manufacturer’s instructions. Subsequently, cells were re-suspended in Williams E Medium supplemented with 0.1 µM DMSO and Cell Maintenance Cocktail B (Life Technologies). Cells were added to collagen-coated plates (Life Technologies), and after 4 h the medium was replaced with fresh culture medium. Cells were incubated at 37 °C for 24 h prior to infection.

### 2.5. RNA Isolation and Sequencing

Viral RNA was extracted from infected CH-SAH cells with TRIzol (Life Technologies) according to manufacturer’s protocol. In order to perform genomic sequencing, complementary DNA (cDNA) was generated following the method outlined by Jiang et al. [30]. Primers were designed to amplify specific viral genes, and the PCR products were sequenced at the University of Guelph Laboratory Services, Guelph, ON, Canada. Pairwise identity of the viral genes and comparison were performed with BLASTn [31].

### 2.6. Viral Growth and Cell Viability Assay

Survival of cancer cell lines after viral infection was determined by PrestoBlue^TM^ Cell Viability Reagent (Life Technologies), a resazurin dye-based metabolic assay. Cells were plated at concentrations of 1 × 10^3^ viable cells/well and allowed to adhere overnight. Cells were either uninfected or infected at various MOIs. At subsequent time points after viral infection, PrestoBlue^TM^ Cell Viability Reagent was added according to the manufacturer’s protocol. Cell viability was determined by comparing fluorescence readings of infected cells to uninfected controls. All samples were run in triplicate for each MOI, and each experiment was performed a minimum of three times.

To assess viral replication, cell monolayers were grown to 80%–90% confluency. Cells in six-well plates were infected with ARV-PB1 at an MOI of 5 for 1 h at room temperature. Subsequently, the inoculum was removed and the cells were washed with phosphate buffered saline (PBS, pH 7.4), and medium was added as described [28]. Cells were harvested at indicated time points and stored at −80 **°**C. Lysates were freeze-thawed three times to release viruses, and the samples were titrated in CH-SAH cells. Each viral growth curve was performed in duplicate.

### 2.7. Cell Staining

Cells were seeded in 35 mm cell culture dishes (5 × 10^5^ cells/dish) containing sterile coverslips. After 24 h incubation at 37 °C, 5% CO_2_, cells were infected with ARV-PB1 (MOI of 5) for 72 h. To study syncytia formation and cytopathic effects as well as to detect the viral genome in infected cells, medium was removed and cells were washed twice with PBS and fixed with 4% buffered-formalin (*v/v*) formalin for 5 min followed by another wash with PBS. Cells were permeabilized with 0.1% NP40-PBS for 10 min at room temperature followed by three washes with PBS. Cells were blocked with5% BSA (bovine serum albumin) in PBS for 1 h at room temperature followed by incubation with anti-dsRNA monoclonal antibody K1 (Scicons, Szirák, Hungary) for 2 h at room temperature. After three washes with PBS, cells were incubated with secondary goat anti-mouse immunoglobulin G (IgG) labeled with Nexa Fluor 594 (Invitrogen, Eugene, OR, USA). After three washes with PBS, cells were stained with 4',6-diamidino-2-phenylindole (DAPI) according to manufacturer’s protocol (Life Technologies). Analysis of cells was performed with a Zeiss Axio Cam MRm fluorescent microscope.

### 2.8. Flow Cytometry

Cells were seeded at a density of 1 × 10^6^ cells per well in a six-well plate, and infected at an MOI of 5 and incubated for 72 h before staining. Medium was removed, and the cells were washed with PBS (pH 7.4) prior to trypsin treatment. Plates were incubated until cells appeared detached. 5 × 10^5^ cells were centrifuged at 1000× *g* for 5 min at room temperature, washed with PBS and stained with Annexin V-FITC (Calbiochem, Billerica, MA, USA) and 7-AAD (eBioscience, San Diego, CA, USA) according to the manufacturer’s protocols. Samples were analyzed by flow cytometry using a FACS Aria IIu with FACSDiva™ Software V6 (BD Biosciences, Mississauga, ON, Canada), while data were analyzed with FlowJo software version X (Tree Star, Ashland, OR, USA).

### 2.9. In Silico Modeling

The nucleotide sequence of the viral S1 gene of ARV-PB1 was analyzed by I-TASSER (http://zhanglab.ccmb.med.umich.edu/I-TASSER/) to predict protein structure and function [32,33]. Based on the generated predictions, we identified the tertiary structure, which most closely resembled the S1 protein—identified as being avian reovirus strain S1133, Protein Data Bank (PDB) ID 2JJL. We next determined the structural similarity between ARV and a previously crystallized mammalian reovirus type 3 S1 protein. The analysis was performed using the mammalian reovirus type 3 (Dearing strain), 1KKE [34] as a reference. Modeling and tertiary alignments were carried out using USCF Chimera (https://www.cgl.ucsf.edu/chimera/).

### 2.10. Plaque Reduction Assays

Plaque reduction assays were performed as described [28] to investigate the presence of neutralizing antibodies in HCC patient sera. Whole blood was collected by venipuncture in a Vacutainer from patients with HCV-induced HCC at the Toronto Western Hospital Liver Clinic (Toronto, ON, Canada). Blood samples were centrifuged and sera stored at −80 °C. The experiments were approved and performed in accordance with the University Health Research Network Research Ethics Board. All patients provided written informed consent for the storage and use of their specimens for the purpose of research (University Health Network Research Ethics Board Biobank Protocol Number 13-6974). Patients tested negative for both human immunodeficiency virus (HIV) and hepatitis B virus (HBV), and were clinically diverse. HCV viral loads ranged from undetectable by the gold standard to 10^4^ IU/mL; genotypes 2, 3, and 4 were represented; patients were either HCV treatment-naïve or experienced, and were either pre- or post-transplant.

### 2.11. Quantitative Polymerase Chain Reaction

Cells seeded in 24-well plates were infected with ARV-PB1 at an MOI of 5. At 6 hours post-infection (h.p.i.), total RNA was extracted using TRI Reagent^®^ (Sigma) according to the manufacturer’s instructions. Isolated RNA was quantified and subjected to DNaseI treatment to degrade genomic DNA. The reverse transcription reactions were performed with an IScript Reverse Transcription Kit (Bio-Rad, Mississauga, ON, Canada) according to the manufacturer’s protocol. Real-time quantitative PCR (qPCR) reactions were carried out for all genes of interest in each sample using Light Cycler 480 SYBR Green1 (Roche Diagnostics, Mississauga, ON, Canada) Gene Expression Assays in a LightCycler 480 II (Roche Diagnostics). The sequences of the PCR primers are listed in Supplementary Table S3. The cycling conditions were: 1 cycle of denaturation at 95 °C for 5 min, followed by 45 three-segment cycles of amplification (95 °C for 10 s, 56–60 °C (gene depending), 72 °C/20 s) where the fluorescence was automatically measured during PCR and one three-segment cycle of product melting (95 °C for 5 s, 65 °C for 60 s, 98 °C continuous mode). The LightCycler480 Relative Quantification Software (Roche Applied Sciences, Penzberg, Germany), was used to determine the threshold cycle (Ct) in each reaction. A melting curve was constructed for each primer pair to verify the presence of one gene-specific peak and the absence of primer dimer. Glyceraldehyde 3-phosphate dehydrogenase (GAPDH) was chosen as the reference housekeeping gene. Each experimental sample was repeated 12 times. For each cDNA sample, the Ct value of each target sequence was subtracted from the Ct value of the reference gene (GAPDH), to derive ΔCt. The level of expression of each target gene, normalized to GAPDH, was then calculated using the delta Ct method, where ΔCt value of each experimental sample was subtracted from ΔCt value of control samples, and log_2_ value was calculated.

### 2.12. Statistical Analyses

Figures were generated using GraphPad Prism 6.0 software (GraphPad Software, Inc., La Jolla, CA, USA). Statistical analyses were also performed using this software. Differences between means were evaluated using the Student’s *t*-test and were deemed significant at *p* ≤ 0.05.

## 3. Results

### 3.1. Isolation and Characterization of ARV-PB1

ARV-PB1 was isolated from a field sample. Virus propagation, titration and one-step growth curves were carried out in chicken hepatoma cells (CH-SAH cell line), as described [28]. ARV-PB1 infection induced cytopathic effect in CH-SAH cells through formation of syncytia, a hallmark of some orthoreoviruses (Figure S1). Reoviruses are known to have segmented genomes. We sequenced the sigma 1 (S1), sigma 2 (S2), sigma 4 (S4) and mu 2 (M2) genomic segments, and analysis by a nucleotide Basic Local Alignment Search Tool (BLASTn) [31] indicated that ARV-PB1 represented an avian orthoreovirus (Table S1).

### 3.2. ARV-PB1 Shows Cytolytic Activity in Cancer Cell Lines

**Figure 1:**
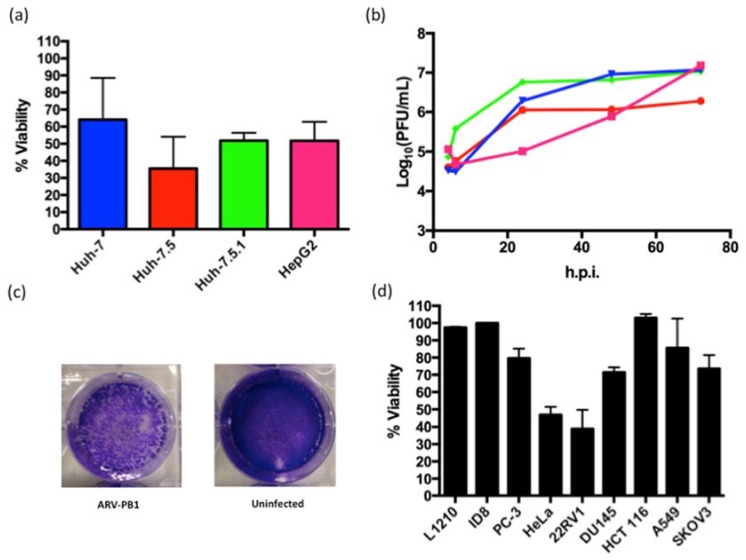
Evaluation of avian orthoreovirus (ARV-PB1) replication in cell lines. (**a**) Cell viability in human liver cell lines was measured at 96 hours post-infection (h.p.i.) with PrestoBlue^TM^ Cell Viability Reagent (Life Technologies) and data was normalized to uninfected controls. Experiments for each cell line were performed a minimum of five times, and error bars represent the standard deviation. (**b**) ARV-PB1 titers at various time points, in cell lines following infection at a multiplicity of infection (MOI) of 5. The experiment was performed in duplicate and data are from one representative experiment. (**c**) Cytopathic effect following crystal violet staining in Huh-7.5 cells at 72 h.p.i. Similar results were observed in the other liver cell lines tested, which included Huh-7, Huh-7.5.1 and HepG2. Experiments were performed in triplicate, and one representative picture is shown. (**d**) Cell viability in other cancer cell lines infected at an MOI of 10 was measured at 96 h.p.i. with PrestoBlue^TM^ Cell Viability Reagent (Life Technologies) and data was normalized to uninfected controls. The origin of the analyzed cell lines is described in Table S2. Experiments for the ID8 cell line was performed twice, all other cell lines were repeated a minimum of three times. Data represent the mean, and error bars represent the standard deviation.

Recent reports have highlighted the potential of reoviruses in oncolytic virotherapy [13]. Moreover, orthoreoviruses can infect a wide range of cells, and avian orthoreoviruses can cause hepatitis in chickens suggesting virus tropism to liver cells [28,21]. These led us to hypothesize that ARV-PB1 could have productive infection and induce cell death in HCC cells. To investigate this, ARV-PB1 was tested against four liver cancer cell lines: Huh-7, Huh-7.5, Huh-7.5.1 and HepG2 (Figure 1a). CPE and a decrease in cell viability were observed upon infection with ARV-PB1. To determine if viral replication was playing a role in oncolysis of HCC cells, one-step growth curves were performed. Each liver cell line supported virus replication with a one-log increase (or greater) over input viral titers, highlighted by a more than two-log increase in HepG2 cells by 72 h.p.i. (Figure 1b). Additionally, immunofluorescent staining of these cells with an antibody against dsRNA indicated the presence of dsRNA within the cells, further confirming active infection (Figure S2). Furthermore, crystal violet staining of infected cells revealed CPE (Figure 1c).

Additionally, we tested the efficacy of ARV-PB1 against a panel of cancer cell lines (Table S2) that represented a range of different tumor types and compared the cell viability between infected and uninfected cells. Slight to moderate decreases in viability were seen in the analyzed cell lines. More extensive decreases in cell viability upon infection were observed in HeLa, and 22Rv1 cells (Figure 1d). We used Reolysin^TM^, an oncolytic human reovirus currently in clinical studies, as a reference virus to compare the cytolytic effects of ARV-PB1 in Huh-7.5 cells. As shown in Figure S3, the decrease of viability of cells infected with ARV-PB1 was comparable to those infected with Reolysin^TM^.

### 3.3. ARV-PB1 Is Not Cytotoxic in Ex Vivo Hepatocytes

**Figure 2:**
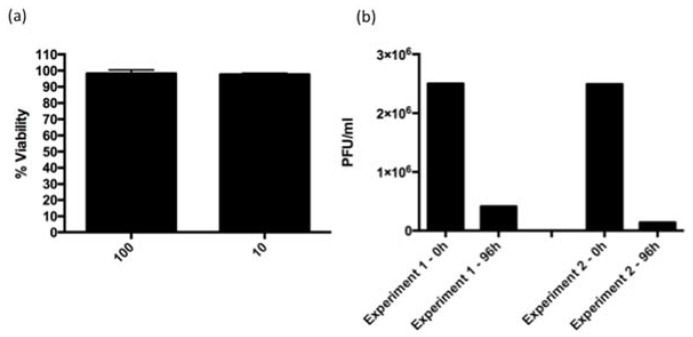
Cytotoxicity assessment of ARV-PB1. (**a**) Primary hepatocytes were infected at multiplicities of infection (MOI) of 10 and 100. After 1 h virus adsorption, the inoculum was removed and cells were washed with phosphate buffered saline (PBS) and fresh medium was added. Cell viability was determined at 96 h.p.i. by PrestoBlue^TM^ Cell Viability Reagent (Life Technologies). Data were normalized to uninfected controls. Experiments were performed four times, and data represent the mean while error bars represent the standard deviation. (**b**) Primary hepatocytes were infected with ARV-PB1 at an MOI of 10 as described above and virus titers were examined at 0 and 96 h.p.i.

An important attribute of OVs is their selectivity for cancer cells. To test the specificity of ARV-PB1 to cancer cells, virus replication and CPE were examined ex vivo in primary hepatocyte cultures from biopsy samples. Upon virus infection, hepatocytes remained viable during the course of the experiment (Figure 2a) and virus titers decreased by 96 h.p.i. relative to the input infection dose (Figure 2b). Collectively, our results suggest that ARV-PB1 replicates and kills HCC cells while sparing normal hepatocytes.

### 3.4. ARV-PB1 Induces Syncytia Formation and Apoptosis in Hepatocellular Carcinoma Cells

**Figure 3:**
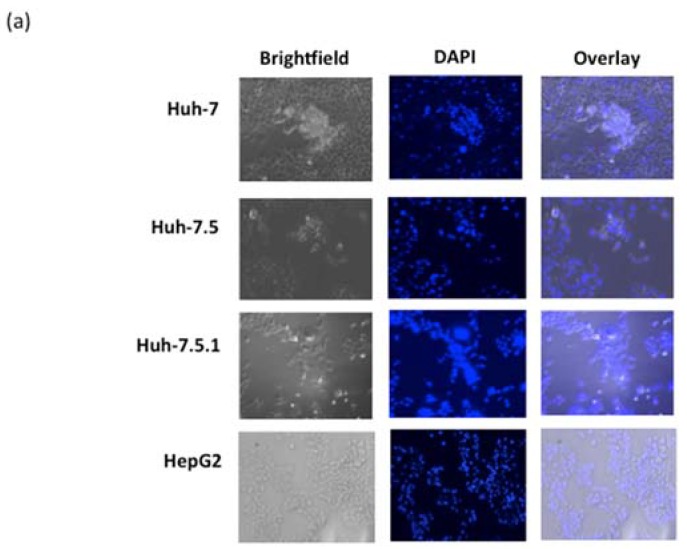

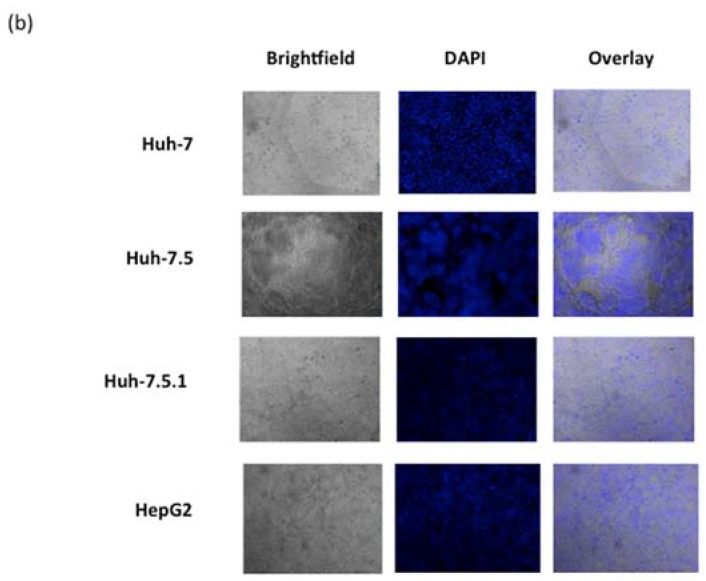
Syncytia formation in liver cell lines. (**a**) Cells were infected at an MOI of 5 for 72 h followed by fixation with formalin and 4',6-diamidino-2-phenylindole (DAPI) staining. (**b**) No syncytial formation was observed in uninfected cells. Cells were analyzed by fluorescence and bright-field microscopy (100× magnification).

The presence of the FAST protein is unique amongst certain members of the family *Reoviridae*, resulting in characteristic formation of syncytia, and induction of apoptosis in infected cells [35,36]. To determine whether infection with ARV-PB1 induced syncytia, liver cells were stained with DAPI allowing visualization of multinucleated cells formed as a result of cell-to-cell fusion (Figure 3). Monoclonal antibody to dsRNA and DAPI staining of the analyzed HCC cell lines confirmed the presence of ARV-PB1 (red fluorescence signals) in multinucleated syncytial cells (Figure S2). Control cells, on the other hand, were negative for the presence of dsRNA and syncytia formation.

**Figure 4:**
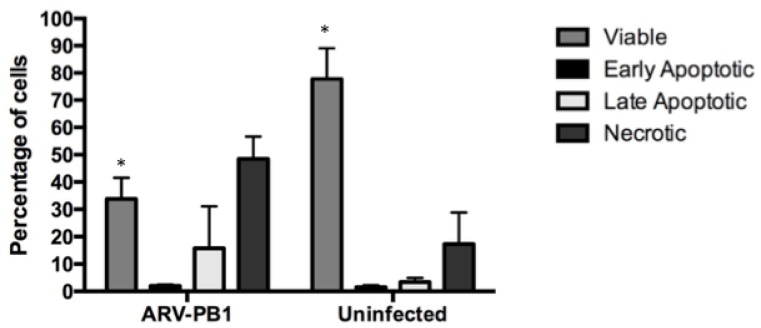
Analysis of apoptosis in hepatocellular carcinoma cells. Huh-7.5 cells were either mock infected or infected with ARV-PB1 at a MOI of 5 for 72 h. Subsequently, mechanisms of cell death were determined by annexin V and 7-AAD staining according to the manufacturer’s instructions. The percentages of viable (annexin V− 7-AAD−), early apoptotic (annexin V+ 7-AAD−), late apoptotic (annexin V+ 7-AAD+) and necrotic (annexin V− 7-AAD+) cells were determined. Data represent the mean from experiments performed in triplicate. Error bars represent standard deviations. Samples were compared using Student’s *t*-test with significant differences indicated by * *p* ≤ 0.05.

Syncytia formation mediated by FAST proteins has also been shown to activate cellular apoptotic pathways [35]. Therefore, to test whether ARV-PB1 induced apoptosis, Huh-7.5.1 cells were stained with annexin V and 7-aminoactinomycin D (7-AAD). During programmed cell death, phosphatidylserine (PS) is translocated to the extracellular membrane, and can be quantified by binding of fluorochrome-labelled annexin V. At 72 h.p.i, there were significantly fewer viable cells amongst the reovirus-infected groups compared to uninfected controls (*p* = 0.005). Furthermore, the percentages of apoptotic and necrotic cells also increased (Figure 4). Similar results were obtained in the other liver cell lines examined in this study (data not shown). These data suggest the correlation between syncytia formation and induction of apoptosis in liver cancer cells.

### 3.5. ARV-PB1 Induces Expression of Interferon-Stimulated Genes

**Figure 5:**
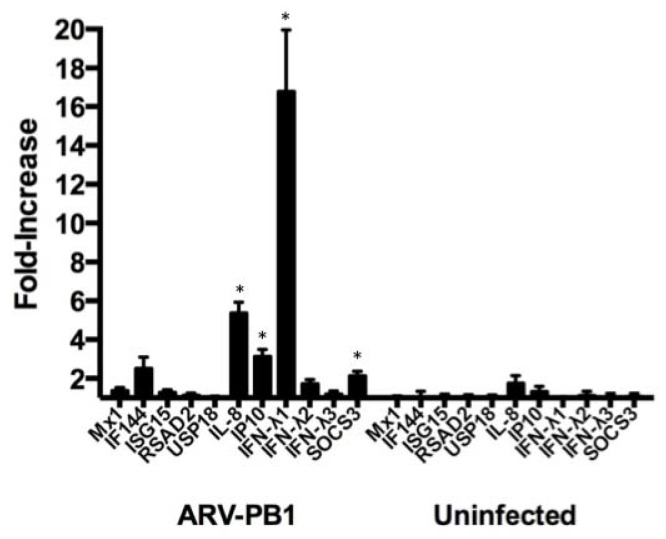
Expression of interferon-stimulated genes (ISGs). Huh-7 cells (5 × 10^5^) were infected at a MOI of 5 and messenger RNA (mRNA) was collected for analysis at 6 hours post-infection. Transcription of the analyzed ISGs was analyzed by quantitative real-time polymerase chain reaction (PCR) with glyceraldehyde 3-phosphate dehydrogenase (GAPDH) as a housekeeping gene and expressed as a fold-change compared to uninfected cells (35). Data represent the mean from six replicate experiments, with error bars showing the standard error of the mean. Samples were compared using Student’s *t*-test with significant differences indicated by * *p* ≤ 0.05.

The induction of antitumor immune responses is important for achieving clearance of tumor cells. Additionally, recent work has shown that the oncolytic effect of OVs is largely determined by the anti-tumor immunity induced upon infection rather than virus-induced cell lysis [7,37,38]. We hypothesized that ARV-PB1 infection would induce the expression of ISGs that are linked to antitumor immunity. To test this hypothesis, Huh-7 cells were infected with ARV-PB1, and we examined the transcription of 11 ISGs involved in the production and induction of interferons at 6 h.p.i. This early time point was selected to ensure that any change in the expression was due to viral infection, rather than autocrine/paracrine effects of interferon production. We considered a >2-fold change in expression to be significant, and this was observed for the genes encoding IF144 (interferon (IFN)-induced protein 44), interleukin (IL)-8, IP10 (10 kDa interferon γ-induced protein), IFN-λ1 and SOCS3 (suppressor of cytokine signaling 3). Although, it was determined that while the expression of *IF144* was elevated, this failed to achieve statistical significance (*p* = 0.06). Notably, *IL8* and *IFN-λ1* showed very high levels of expression, with 5.3- and 16.7-fold increases, respectively (Figure 5).

### 3.6. Activity in Hepatitis C Virus Replicon-Containing Cells

**Figure 6:**
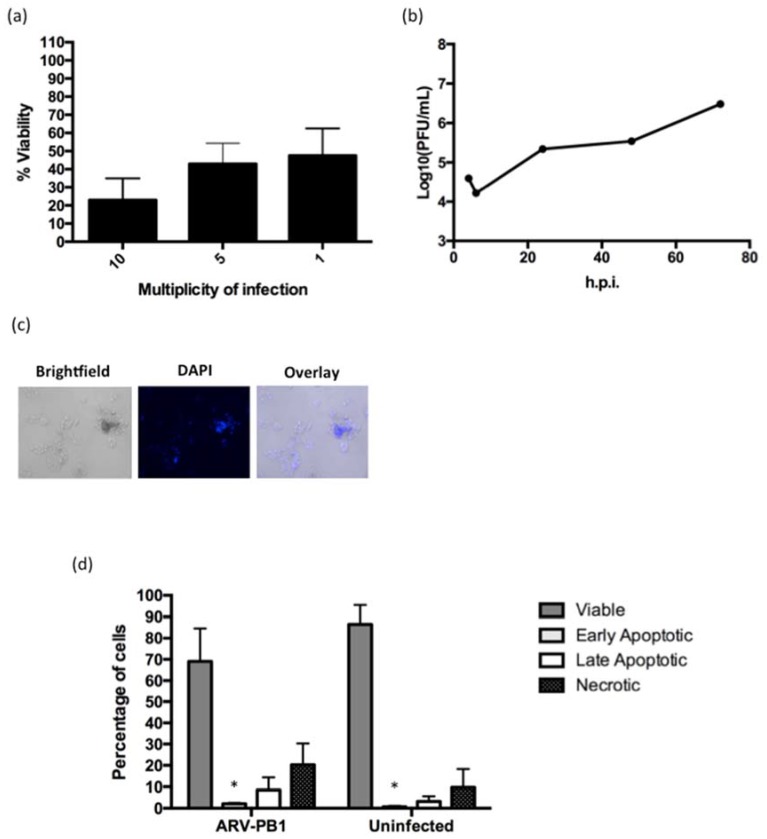
ARV-PB1 activity in HCV-replicon-containing cells. (**a**) Cell viability in BB7 cells infected at differing MOIs was measured at 96 hours post-infection (h.p.i.) using PrestoBlue^TM^ Cell Viability Reagent (Life Technologies). Data were normalized to uninfected controls. The experiment was performed at three times, and error bars represent the standard error of the mean. (**b**) ARV-PB1 viral titers in BB7 cells at various time points following infection at a MOI of 5. (**c**) Fluorescent and bright-field microscopy (100× magnification) to view DAPI staining and evaluate syncytia in BB7 cells infected with ARV-PB1 (MOI of 5) at 96 h.p.i. The experiment was performed in triplicate and data are from one representative experiment. (**d**) BB7 cells were either uninfected or infected with ARV-PB1 (MOI of 5) for three days. Subsequently, the cells were fixed and treated with annexin V and 7-AAD. The percentages of viable (annexin V− 7-AAD−), early apoptotic (annexin V+ 7-AAD−), late apoptotic (annexin V+ 7-AAD+) and necrotic (annexin V− 7-AAD+) cells were determined. Data represent the means from experiments performed in triplicate. Error bars show standard deviations. Samples were compared using Student’s *t*-test with significant differences indicated by * *p* ≤ 0.05.

Infection with HCV is a major cause of HCC, and recurrent infection can occur even after liver transplantation [2]. Therefore, we investigated whether ARV-PB1 had cytolytic effect in an in vitro model of infection with HCV by using BB7 cells, a cell line derived from Huh-7.5.1 cells, which stably contains the HCV subgenomic genotype 1a H77 replicon. As shown in Figure 6, cell viability decreased as the MOI increased, and productive infection and titers were comparable to those in other liver cell lines. Likewise, virus infection induced syncytia and apoptosis in BB7 cells (Figure 6). Additionally, the decrease in cell viability upon infection with ARV-PB1 was comparable to that with Reolysin^TM^ (Figure S3). Our results suggest that ARV-PB1 has cytolytic activity in HCC cell lines containing the HCV replicon, and that the presence of HCV does not prevent replication of ARV-PB1.

### 3.7. ARV-PB1 Shows Cytolytic Activity in JFH-1 Infected Cells

**Figure 7:**
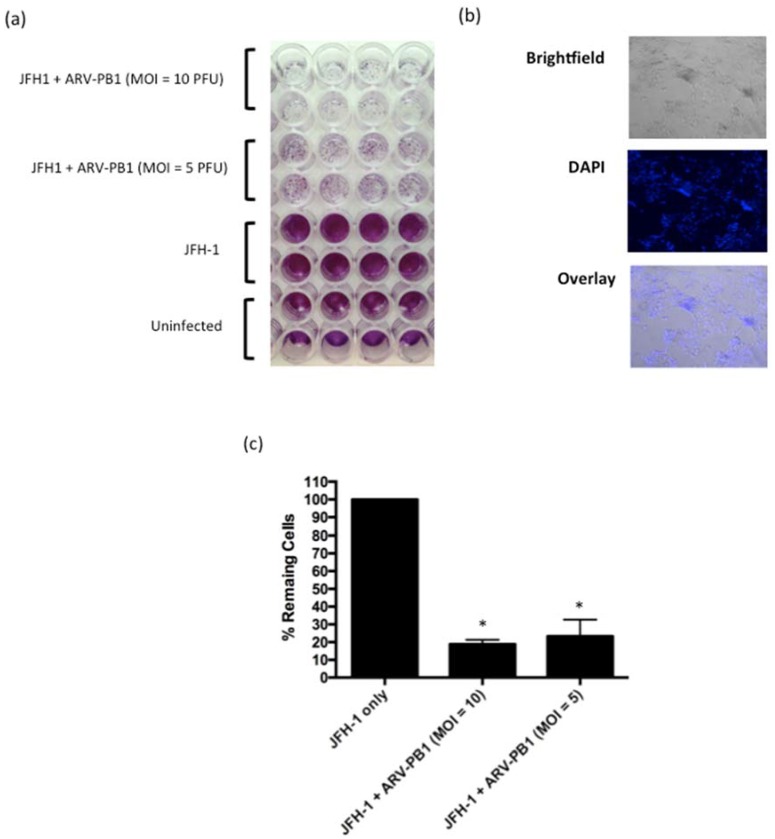
ARV-PB1 activity in JFH-1 -infected cells. (**a**) Huh-7.5.1 cells were infected with JFH-1 and 24 h later treated with ARV-PB1 at differing MOIs. At 48 hours post-treatment, cells were fixed and stained with crystal violet to evaluate cytopathic effect. The experiment was performed in triplicate. (**b**) Induction of syncytia in JFH-1-infected cells by ARV-PB1. Cells were infected with ARV-PB1 (MOI of 5) for 48 h. Subsequently, cells were fixed with formalin, stained with DAPI and analyzed by fluorescent and bright-field microscopy (100× magnification). (**c**) Measurement of remaining viable cells following treatment with various MOIs at 48h post-infection. The experiment was performed at four times, and error bars represent the standard deviation. Samples were compared using Student’s *t*-test with significant differences indicated by * *p* ≤ 0.05.

The HCV genotype 1a H77 replicon contains only non-structural genes and thus virus replication and spread are ablated [39]. Therefore, we also tested ARV-PB1 against Huh-7 and Huh-7.5.1 cells infected with the lab-adapted infectious virus strain JFH-1. This virus, unlike the H77 replicon, contains the complete HCV genome, and is capable of producing infectious particles, making it a more physiologically relevant model of HCV infection [40]. The effect of ARV-PB1 on the viability of JFH-1-infected cells was assessed at 48 h.p.i. CPE was observed upon infection with ARV-PB1 while absent in either uninfected cells or those infected with JFH-1 (Figure 7a,c). Moreover, DAPI staining showed large multinucleated cells and syncytia formation (Figure 7b), similar to those that were seen in cell lines containing the HCV replicon. This further demonstrates that infection with HCV does not inhibit replication and spread of ARV-PB1.

### 3.8 Serum Neutralization and In Silico Modeling

Neutralizing antibodies can limit the systemic distribution and therapeutic potential of OVs [15]. Mammalian reovirus type 1 σ1 adhesin fiber induces neutralizing antibodies [41]. In order to investigate whether antibodies to mammalian orthoreovirus would neutralize ARV-PB1, we performed plaque reduction assays using sera from patients with HCV-induced HCC and healthy volunteers. Sera from both HCC patients and healthy volunteers failed to reduce the number of virus induced plaques, which suggests the lack of pre-existing immunity to ARV-PB1

**Figure 8:**
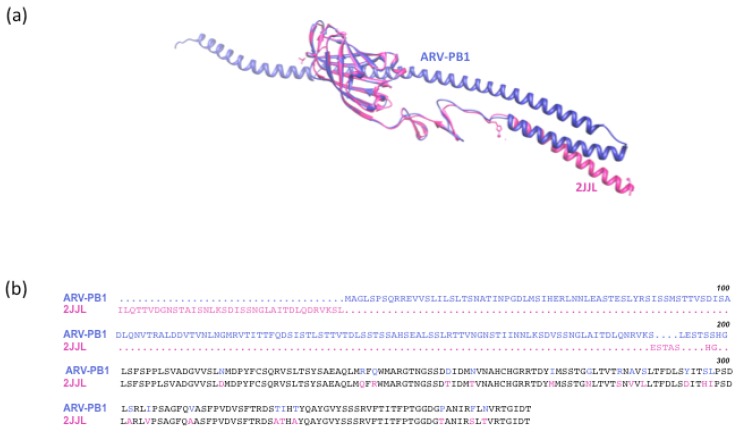

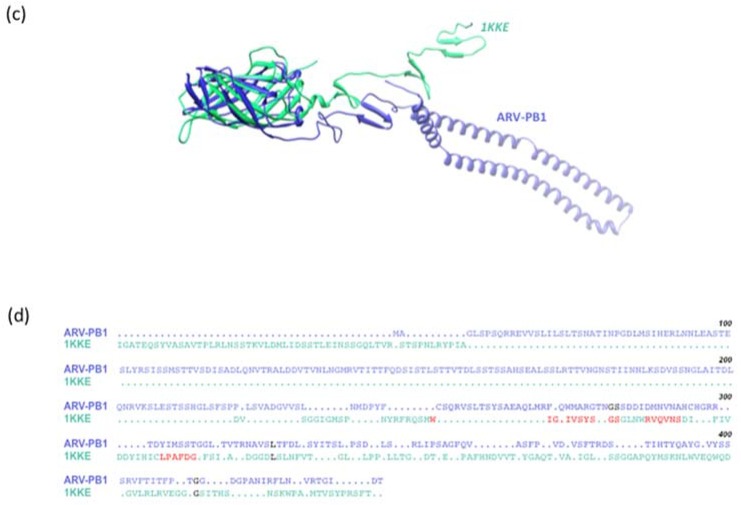
In silico structure generation of the S1 attachment protein of ARV-PB1. (**a**) I-TASSER-generated ARV-PB1 (blue), manually superimposed with avian reovirus σ C117-326, Protein Data Bank (PDB) ID 2JJL (pink), identified as the most closely related crystal structure. (**b**) Tertiary structural alignment of the S1 protein of ARV-PB1 and 2JJL. Conserved sequence regions are highlighted in black. (**c**) I-TASSER-generated ARV-PB1 (blue), manually superimposed with mammalian orthoreovirus 3 (Dearing strain), PDB ID 1KKE (green), using 1KKE as a generation template. (**d**) Tertiary structural alignment of the S1 protein of ARV-PB1 and 1KKE. Conserved sequence regions are highlighted in black, and the epitope targets of neutralizing antibodies are highlighted in red.

Using the online server I-TASSER we first analyzed the S1 gene to determine the most closely related crystallized structure. Although truncated, the server identified a structure of avian reovirus S1133 fiber confirming the identity of ARV-PB1 (Figure 8b). As demonstrated, the β-pleated sheets within this protein overlap with sequence homology of 87% in this region. Next, we used mammalian orthoreovirus type 3 (Dearing strain) as a reference to assess the tertiary structural similarity between human and avian orthoreovirus adhesion fibers. There is much less structural homology with the human protein in comparison to the crystallized avian protein, as evidenced by the minimal homology in beta-pleated sheets, and structural sequence alignments (Figure 8c,d). Furthermore, the structural alignment revealed significant amino acid difference in the epitope associated with antibody binding (highlighted in red) [41,42], suggesting that antibodies generated in patients who had received mammalian orthoreovirus type 3 (Dearing strain) (Reolysin^TM^) would not neutralize ARV-PB1.

## 4. Discussion

The anticipated increase in worldwide HCC burden, combined with the poor outcomes, highlights the urgent need for new therapeutic options. We have isolated an avian orthoreovirus, ARV-PB1, and demonstrated its cytolytic activity in vitro against several liver cancer cell lines, including those infected with HCV. Therefore, our results suggest the oncolytic potential of ARV-PB1. Our results also suggest that the mechanisms of ARV-PB1-induced apoptosis are likely through syncytia formation by FAST proteins, as known for fusogenic reoviruses [36]. Interestingly, the presence of HCV did not impede viral replication, nor did it influence the oncolytic effects of ARV-PB1. Additionally, ARV-PB1 was an inducer of ISGs, which may have contributed to the apoptosis in Huh-7 cells. Therefore, we speculate that cytolytic properties of ARV-PB1 are likely determined by combination of direct cytopathic and immunostimulatory effects.

Recent clinical trials have highlighted the therapeutic potential of OVs, particularly reoviruses [9,13,23,42,43]. ARV-PB1 may provide a means of overcoming the challenges of pre-existing immunity that may otherwise limit the applicability of some of the identified OVs. The *σ1* gene encodes the adhesin protein, which is the target of neutralizing antibodies [41,42]. It was noted that ARV-PB1 was not neutralized by a commercially available S1 adhesion protein-specific antibody, and that neutralizing antibodies against ARV-PB1 were not present in the serum of HCC patients infected with HCV. Further, significant structural and tertiary alignment sequence differences were found between the ARV-PB1 σ1 and its homolog in the mammalian reovirus serotype 3. Therefore, the presence of neutralizing antibodies in individuals treated with mammalian reoviruses (Reolysin^TM^) would be low, and unlikely to inhibit ARV-PB1. Therefore, there is a potential for synergy with Reolysin^TM^, or other OVs, or in combination with chemo drugs. Vaccinia virus and vesicular stomatitis virus are known to synergize in various tumor models [42]. Therefore, the use of ARV-PB1 in conjunction with another OV effective against HCC (such as JX-594 [38,44]) would be worth investigating.

This is the first report of the cytolytic effects of an avian reovirus against HCC, suggesting the potential oncolytic properties of ARV-PB1. We also demonstrated that ARV-PB1 neither replicates nor induces CPE in primary hepatocytes isolated from biopsy samples. Although treatments for HCV are rapidly improving and becoming more available, HCV continues to be a leading cause of liver cancer worldwide. To date, other OVs targeting HCC have shown efficacy against HBV and HCV-associated HCC [37,45]. Interestingly, recent work by Samson and colleagues has demonstrated the in vitro and in vivo oncolytic activity of a mammalian reovirus against HCC, including the ability to eradicate HCV from cells [45]. Moreover, induction of interferon was an important component of the antitumor response, as an effect was also seen with UV-inactivated virus. These findings support our data as we noted induction of IFN-λ1 as well oncolytic effect in HCV-infected cell lines. Future experiments will be performed to investigate if the oncolytic effect observed with ARV-PB1 is altered following UV-inactivation. However, it is tempting to speculate that the combination therapy with both mammalian and avian reoviruses could result in enhanced efficacy.

It has been shown that HCV inhibits protein kinase R (PKR), thereby preventing the induction of the antiviral state, which could promote replication of other viruses [46,47]. Additionally, some evidence suggests that inactive PKR may be unable to phosphorylate p53 and may also play a role in tumorigenesis through activation of signaling pathways involved in cell proliferation [47,48]. Therefore, activation of PKR may help in creating an anti-tumor environment in cells. Recent work demonstrated that ARVs may preferentially activate PKR and AMPK (AMP-activated protein kinase) as a means to aid viral replication [27,48]. Thus,we speculate that the CPE observed in cells co-infected with JFH-1 and ARV-PB1, may be due to HCV-mediated inhibition of PKR creating a favorable cellular environment for ARV-PB1 replication.

Our study demonstrated the ability of ARV-PB1 to form syncytia, and correlated this with the induction of apoptosis. Additionally, the sequence analysis revealed a putative homolog to the p10 gene found in ARV-138, known to encode a FAST protein (data not shown). It has been shown that genes encoding this family of proteins are found in some members of the family *Reoviridae*; however, they are not present in the mammalian reovirus currently being investigated as a virotherapeutic [35]. Moreover, it has been noted that vesicular stomatitis virus expressing the p14 FAST protein is able to induce cell fusion, and enhanced production of another oncolytic virus when administered together [43]. Thus, we hypothesize that the presence of a predicted FAST protein in ARV-PB1 will promote enhanced viral spread and oncolytic effect within the tumor environment, and we intend to test this in animal models in future studies.

An important goal of OV therapy is to achieve intratumoral spread to debulk the tumor while concurrently inducing an innate immune response. This assists in the removal of remaining tumor cells by direct mechanisms, or by activating adaptive immunity. As illustrated by our results, ARV-PB1 induced higher expression of IFN-λ1. This cytokine has been associated with induction of apoptosis and natural killer (NK) cell recruitment in the BNL cell line based model of HCC [49]. Three requirements have recently been highlighted for an OV to achieve a viroimmunotherapy response [50]: selectivity for cancer cells, induction of a potent immune response, and exposure of tumor-associated antigens to the immune system. This report demonstrates that ARV-PB1 selectively targets cancer cells, and induces genes associated with a potent innate immune response. We have proposed several mechanisms to explain its oncolytic activity in vitro. Taken together, these data provide rationale for in vivo tumor models to study the oncolytic effects of ARV-PB1.

## Supplementary Materials

The following are available online at www.mdpi.com/1999-4915/9/4/90/s1, Figure S1: Cytopathic effect in response to viral infection, Figure S2: Presence of viral RNA in infected cells, Figure S3: Comparison of ARV-PB1 to Reolysin^TM^ in Huh-7.5 cells, Figure S4: Comparison of ARV-PB1 to Reolysin^TM^ in BB7 cells, Table S1: Homology of ARV-PB1 nucleotide sequences, Table S2: Characteristics of cancer cell lines, Table S3: Primers for qPCR.
