# Supplementary material for: Replication and Oncolytic Activity of an Avian Orthoreovirus in Human Hepatocellular Carcinoma Cells
Source: Viruses. 2017 Apr 24;9(4):90. doi: 10.3390/v9040090 (PMC5408696; doi:10.3390/v9040090)
Supplement: Supplementary file 1 [file viruses-09-00090-s001.pdf]

Replication and Oncolytic Activity of an Avian Orthoreovirus in Human Hepatocellular Carcinoma Cells

Robert A. Kozak, Larissa Hattin, Mia J. Biondi, Juan C. Corredor, Scott Walsh, Max Xue-Zhong, Justin Manuel, Ian D. McGilvray, Jason Morgenstern, Evan Lusty, Vera Cherepanov, Betty-Anne McBey, David Leishman, Jordan J. Feld, Byram Bridle and Éva Nagy

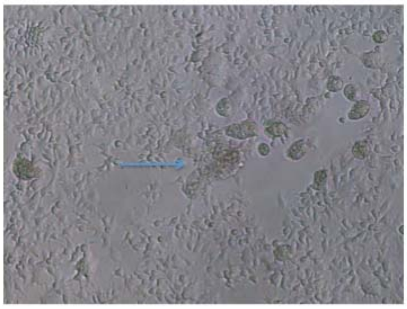

Figure S1. Cytopathic effect in response to viral infection. CH-SAH chicken cells were infected at a MOI of 0.5 for 24 h. The arrow indicates the syncytia.

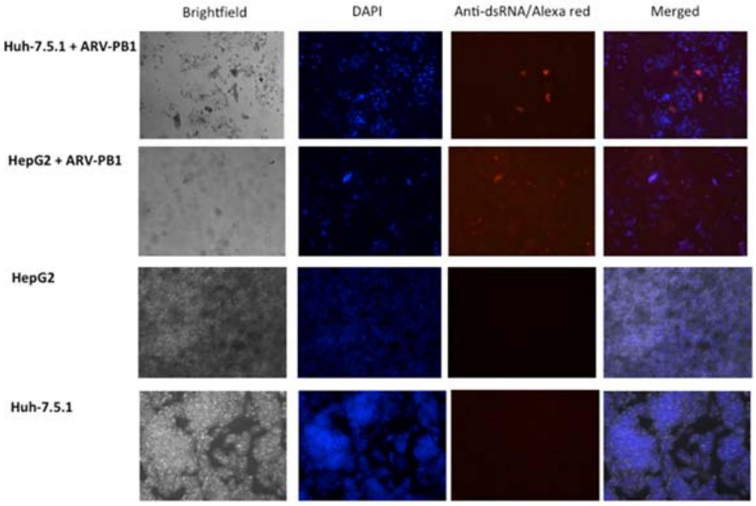

Figure S2. Presence of viral RNA in infected cells. Cells were infected with ARV-PB1 (MOI of 5) for 48 h and then were fixed with formalin, stained with DAPI and an anti-dsRNA monoclonal antibody, and analyzed by fluorescent and bright-field microscopy (100× magnification). No dsRNA was detected in uninfected cells.

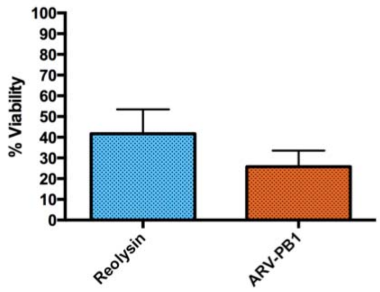

Figure S3. Comparison of ARV-PB1 to Reolysin™ in Huh-7.5 cells. Cells were infected with ARV-PB1 (MOI of 10) or Reolysin™ (MOI of 10) for 96 h. Viability was determined by PrestoBlue™ Cell Viability Reagent and data were normalized to uninfected controls. The experiment was performed in triplicate, mean was calculated, and error bars represent the standard error of the mean.

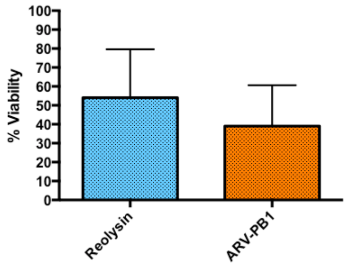

Figure S4. Comparison of ARV-PB1 to Reolysin™ in BB7 cells. Cells were infected with ARV-PB1 (MOI of 10) or Reolysin™ (MOI of 10) for 96 h. Viability was determined by PrestoBlue™ Cell Viability Reagent and data was normalized to uninfected controls. The experiment was performed in triplicate, mean was calculated, and error bars represent the standard error of the mean.

Table S1. Homology of ARV-PB1 nucleotide sequences.

Segment	Homologue	% Pairwise identity
S1	Avian orthoreovirus clone 138 (Accession No.: AF218359)	90
S2	Avian orthoreovirus strain GX110058 (Accession No.: KF741743.1)	92
S4	Avian orthoreovirus strain AVS-B (Accession No.: FR694200)	94
M2	Avian orthoreovirus clone 526 (Accession No.: KF741700)	94

Table S2. Characteristics of cancer cell lines.

Cells	Origin	Tissue	Disease
Huh-7	Human	Liver	Hepatocellular carcinoma
Huh-7.5	Human	Liver	Hepatocellular carcinoma
Huh-7.5.1	Human	Liver	Hepatocellular carcinoma
HepG2	Human	Liver	Hepatocellular carcinoma
DU-145	Human	Prostate	Carcinoma
PC-3	Human	Prostate	Adenocarcinoma
22Rv1	Human	Prostate	Carcinoma
SKOV-3	Human	Ovary	Adenocarcinoma
HeLa	Human	Cervix	Adenocarcinoma
HCT 116	Human	Colon	Colorectal carcinoma
L1210	Murine	Lymphoblast	Lymphocytic leukemia
ID8	Murine	Ovary	Neoplasm-containing carcinoma and sarcomatous components
A549	Human	Lung	Carcinoma

Table S3. Primers for qPCR.

Gene	Forward primer	Reverse primer
GAPDH	5′-TGCACCACCAACTGCTTAGC-3′	5′-GGCATGGACTGTGGTCATGAG-3′
USP18	5′-CAGACCCTGACAATCCACCT-3′	5′-AGCTCATACTGCCCTCCAGA-3′
RSAD2	5′-GTGGTTCCATTATGGTGAGTATTT-3′	5′-CCACGGCCAATAAGGACATT-3′
Mx	5′-CAGCACCTGATGGCCTATCA-3′	5′-ACGTCTGGAGCATGAAGAACTG-3′
ISG15	5′-GAG AGG CAG CGA ACT CAT CT-3′	5′-CTT CAG CTC TGA CAC CGA CA-3′
IF144	5′-TGCTCTTTCTGACATCTCGGTGGT	5′-AGAAGTCATCTGCAGCCCATAGCA-3′
STAT1	5′-CCATCCTTTGGTACAACATGC-3′	5′-TGCACATGGTGGAGTCAGG-3′
IP10	5′-GTACGCTGTACCTGCATCAGCATT-3′	5′-GGATTCAGACATCTCTTCTCACCC-3′
SOCS3	5′-GGCCACTCTTCAGCATCTC-3′	5′-ATCGTACTGGTCCAGGAACTC-3′
IL-29	5′-CACAGGAGCTAGCGAGCTTCA-3′	5′-TTTTCAGCTTGAGTGACTCTTCCA-3′
IL-28A	5′-GACCCAGCCCTGGTGGAC-3′	5′-CTGGATACAGGCCCGGAA-3′
IL-28B	5′-GGCCTTTAAGAGGGCCAAAG-3′	5′-GGCGGGAGCGGCACT-3′
IL-8	5′-TGCCAAGGAGTGCTAAAG-3′	5′-CTCCACAACCCTCTGCAC-3′

© 2017 by the authors. Licensee MDPI, Basel, Switzerland. This article is an open access article distributed under the terms and conditions of the Creative Commons Attribution (CC BY) license (http://creativecommons.org/licenses/by/4.0/).
